# Comprehensive Quantitative Assessment of Lung Liquid Clearance by Lung Ultrasound Score in Neonates with No Lung Disease during the First 24 Hours

**DOI:** 10.1155/2020/6598348

**Published:** 2020-02-24

**Authors:** Bin-Bin Guo, Kun-Kun Wang, Li Xie, Xiu-Juan Liu, Xiao-Ya Chen, Feng Zhang, Chuang Chen, Chang-Jun Wu

**Affiliations:** ^1^Department of Ultrasound, First Affiliated Hospital of Harbin Medical University, Harbin 150001, China; ^2^Department of Computer Tomography, First Affiliated Hospital of Harbin Medical University, Harbin 150001, China

## Abstract

**Objectives:**

To comprehensively and quantitatively assess the process of lung liquid clearance using the lung ultrasound score. This study is to evaluate the whole healthy lungs of neonates during the first 24 h.

**Methods:**

Lung ultrasound was performed in neonates with no respiratory symptoms within 3 h after birth, and scans were then repeated at 6 hours and 24 hours, respectively. The entire chest wall was divided into 12 regions. The lung ultrasound scores of the anterior, posterior, upper, and lower regions and sum of all regions were calculated according to the ultrasound pattern of each region examined.

**Results:**

The total lung ultrasound score decreased gradually during the first 24 h, with the total lung ultrasound score at 6 h being significantly lower than that at <3 h (*P* < 0.05). At <3 h, B-lines were more abundant in the posterior chest than in the anterior chest (*P* < 0.05). At <3 h, B-lines were more abundant in the posterior chest than in the anterior chest (*P* < 0.05). At <3 h, B-lines were more abundant in the posterior chest than in the anterior chest (

**Conclusion:**

Changes in the lung ultrasound score may quantitatively reflect the characteristics of different regions and processes of lung liquid clearance during the first 24 h.

## 1. Introduction

The lungs are vital and vulnerable organs in neonates; therefore, assessment of the lungs is a major concern of neonatologists [1]. Traditional tools include computed tomography (CT) and chest radiographs, which both expose neonates to potentially harmful radiation [2, 3]. Compared with these modalities, lung ultrasound (LUS) is nonirradiating and easily repeatable with no side effects and provides accurate diagnostic information in real time [4, 5].

Lung ultrasound has been increasingly used to differentiate and diagnose neonatal diseases causing respiratory symptoms as a useful bedside diagnostic tool [6–16]. However, few studies have addressed the ultrasound findings of normal lungs in neonates; in the most cases, these findings have only been described qualitatively [17, 18]. To the best of our knowledge, only one study has quantitatively monitored lung liquid clearance in healthy neonates [19], but the results may not be sufficiently comprehensive. The aim of this study was to comprehensively and quantitatively assess lung liquid clearance using the lung ultrasound score in neonates with no lung disease during the first 24 h.

After birth, the neonate gas exchange transitions from dependency on the placenta to dependency on the lungs, and the process must involve airway liquid clearance and air entry into the lungs [17, 18], which results in fluid accumulation in the alveolar interstitial space and then generates lung ultrasound vertical artifacts called B-lines [18, 20]. One animal study showed a good correlation between lung liquid evaluated using gravimetry and that evaluated using B-lines by lung ultrasound [21], and another one noted that the B-line score determined by LUS was quite valuable as a semiquantitative index [22]. In addition, B-lines have been found in both pathological and normal conditions at the lung surface [23, 24]. Therefore, it is reasonable to hypothesize that changes in B-lines could be used to monitor changes in the lung liquid in the healthy lungs of neonates. The current study was designed to quantitatively estimate scores according to the amounts of B-lines to characterize the process of airway liquid clearance in neonates in the first 24 h after birth.

## 2. Methods

### 2.1. Study Design

This was a prospective study of neonates. The study was approved by the First Affiliated Hospital of Harbin Medical University Human Research Ethics Committee.

We studied neonates with no respiratory symptoms born in the First Affiliated Hospital of Harbin Medical University from May 2015 to September 2018. Informed written consent was obtained from their parents. First lung ultrasound video recordings of neonates were obtained no later than 3 hours after birth, and scans were then repeated at 6 hours and 24 hours after birth, respectively. Neonates diagnosed pulmonary diseases before discharge from hospital and neonates with congenital heart disease were excluded from the study.

Epidemiological data were collected, including Apgar scores, mode of delivery, sex, birth weight, gestational age, and clinical diagnosis. According to delivery mode, the neonates were divided into a cesarean section group and a vaginal delivery group.

### 2.2. Lung Ultrasound

**Figure 1:**
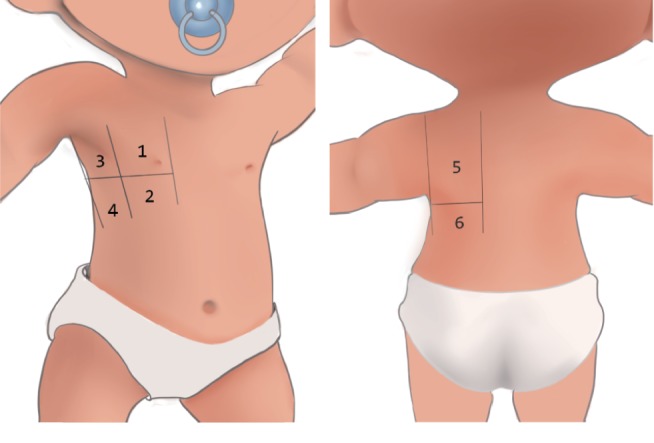
Lung ultrasound regions. The anterior and posterior axillary lines as practical landmarks define the anterior, lateral, and posterior areas of both lungs.

Serial LUS video recordings were obtained using MyLab30, Esaote, Genova, Italy, with a 3- to 11-MHz linear transducer. As previously recommended [25, 26], the chest wall was divided into 12 regions. We delineated the anterior and posterior axillary lines as practical landmarks that defined the anterior, lateral, and posterior areas of both lungs (6 regions per side, Figure 1). All 12 intercostal regions were examined extensively with the neonates in a supine, lateral, and prone position, and one video clip was obtained for each region. We collected these stored videos from the machine.

**Figure 2:**
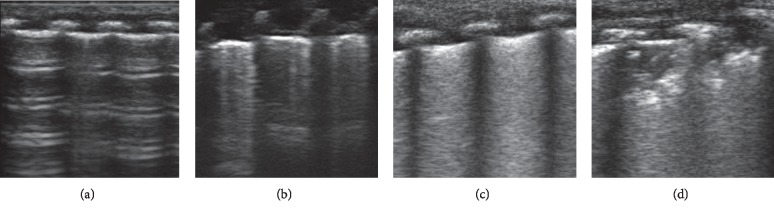
Four ultrasound aeration patterns. (a) Normal aeration (N): the presence of lung sliding with only A-lines or fewer than three isolated B-lines; (b) moderate aeration loss: interstitial syndrome, localized pulmonary edema, defined by coalescent B-lines in less than 50% of the intercostals space, multiple well-defined B-lines, or subpleural consolidations; (c) severe aeration loss: alveolar edema, defined by diffused coalescent B-lines occupying the whole intercostals spaces; (d) complete aeration loss (C): the presence of a tissue pattern with or without air bronchogram.

Four ultrasound aeration patterns were defined: (1) normal aeration (N): the presence of lung sliding with only A-lines or fewer than three isolated B-lines; (2) moderate aeration loss: interstitial syndrome, localized pulmonary edema, defined by coalescent B-lines in less than 50% of the intercostals space, multiple well-defined B-lines, or subpleural consolidations (B1-lines); (3) severe aeration loss: alveolar edema, defined by diffused coalescent B-lines occupying the whole intercostals spaces (B2-lines); and (4) complete aeration loss (C): the presence of a tissue pattern with or without air bronchogram (Figure 2). LUS scores were correspondingly defined as follows: *N* = 0, *B*1 = 1, *B*2 = 2, and *C* = 3. We considered the worst ultrasound abnormality detected as the characterizing region examined [27]. The LUS scores of anterior, posterior, upper, and lower regions and sum of all regions were calculated according to the ultrasound pattern of each area examined. Two blinded observers independently evaluated all recordings.

### 2.3. Statistical Analysis

All statistical analyses were performed using SAS 9.3 international standard statistical programming software. The data were described by the median and upper and lower quartiles, and the Wilcoxon rank-sum test was used for the comparison of paired and independent samples. An intraclass correlation coefficient was used to calculate interrater reliability. Statistical significance was defined as *P* < 0.05.

## 3. Results

### 3.1. General Characteristics

Sixty-six neonates were enrolled, and we studied 2376 videos. The time of examinations was 144.5 (102–163) min, 363 (352–381) min, and 26 (25–27) hours after birth, respectively. B-lines were observed in most neonates (59/66) within 3 h after birth and in 39/66 at 6 h and 30/66 at 24 h after birth. None of the neonates had any region of complete aeration loss throughout the parenchyma during the first 24 h. There were 34 cases of cesarean section and 32 cases of vaginal delivery. The clinical data of 66 neonates are shown in Table 1.

### 3.2. Total Scores

The total lung ultrasound score decreased gradually in neonates during the first 24 h (Table 2).

### 3.3. Regional Scores

**Figure 3:**
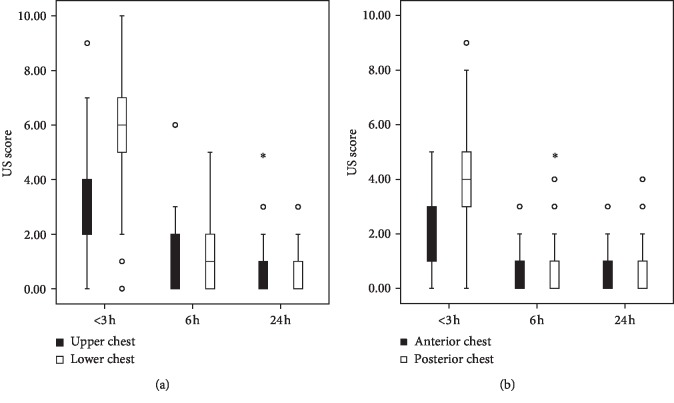
(a) Lung ultrasound scores at <3 h, 6 h, and 24 h postnatally in the upper and lower chest. (b) Lung ultrasound scores in the anterior and posterior chest at <3 h, 6 h, and 24 h postnatally.

At <3 h, there were significant differences in the number of B-lines among the regions. B-lines were more abundant in the posterior chest than in the anterior chest (*P* < 0.001; Figure 3(a)). There were significantly more B-lines in the lower chest than in the upper chest (*P* < 0.001; Figure 3(b)).

At 6 h and 24 h, there was no significant difference between the posterior and anterior chest and between the upper and lower chest (Figure 3).

### 3.4. Scores by Delivery Mode

**Figure 4:**
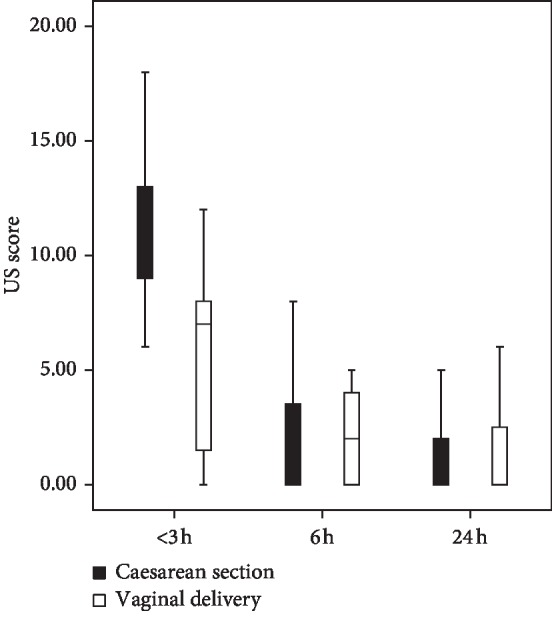
Lung ultrasound scores at <3 h, 6 h, and 24 h postnatally by delivery mode.

We also compared the B-line score between neonates delivered vaginally and those delivered by cesarean section. There were nonsignificant differences in the postnatal age at measurement, demographic data, and clinical characteristics between the two groups (*P* > 0.05). At <3 h, there were significantly more B-lines in neonates delivered by cesarean section than in those delivered vaginally (*P* < 0.001; Figure 4). At 6 h and 24 h, there were no significant differences in the amounts of B-lines between the two groups.

### 3.5. Interrater Consistency

There was a good correlation between the measurements of the two observers. (*r* = 0.833 at <3 h, *r* = 0.816 at 6 h, and *r* = 0.829 at 24 h).

## 4. Discussion

In this study, we show a comprehensive quantitative assessment of lung liquid clearance by the lung ultrasound score in neonates without lung disease during the first 24 h. Compared with another quantitative trial of healthy neonates [19], our study examined the whole lungs, including not only the anterior and lateral chest but also the posterior chest. Moreover, our results show that B-lines were more abundant in the posterior chest than in the anterior chest at <3 h. Although there were less significant differences at 6 h and 24 h, the B-line scores of the posterior chest were still higher than those of the anterior chest. We consider that the distribution of redundant liquid may be attributed to gravity. Although our findings support the premise that the neonates had healthy lungs, whether these results apply to newborns with injured lungs remains to be studied. In fact, one study has shown that ventilation in the prone position can result in a gradient decrease in the gravity distribution of intrathoracic pressure [28], and a review has shown that the prone position can improve oxygenation in neonates undergoing mechanical ventilation compared to the supine position [29]. The results of current and previous studies suggest that the posterior chest is an important part to study in airway liquid clearance, the process of which requires comprehensive assessment.

We observed that the total lung ultrasound score, as expected, decreased gradually in neonates without lung disease during the first 24 h. These results suggest that changes in B-lines are consistent with the physiological process of airway liquid clearance in neonates without lung disease, which is also in agreement with previous research [19].

In the current study, more B-lines were observed in the lower chest than in the upper chest at <3 h. A similar observation was reported by Laura Martelius, who suggested that airway liquid clearance may occur later in the lower lobes than in the upper lobes [19]. Others have shown that in preterm rabbit pups, initiating ventilation at birth with 0 PEEP could result in the unequal distribution of air between the upper and lower lobes, with a significantly greater distribution of air toward the upper than the lower lobes at functional residual capacity [30].

We found at <3 h, there were more B-lines in neonates born by cesarean section than in those born vaginally. A similar observation was reported by Laura et al. [31], who demonstrated that vaginal delivery was associated with a significantly lower lung liquid content than cesarean section at 3 h after birth.

Our study had several limitations. First, the subjects of our study were term infants and late preterm infants, so it is not known whether our results are applicable to other gestational ages. In fact, most early preterm infants are treated prophylactically with pulmonary surfactant after birth, which makes it impossible to objectively observe the process of airway liquid clearance. Second, although the accuracy of the results increases with the inclusion of more sectors, the analysis of more sectors is also more time consuming and not quite suitable for clinical application. Thus, we will seek a comprehensive and efficient method for assessing lung liquid clearance. Third, in this study, the examination time interval was slightly long, which may have resulted in the exact cut-off time when lung liquid clearance is achieved being missed. In future studies, we will increase the examination frequency appropriately.

## 5. Conclusion

This was a comprehensive study using a 12-sector LUS approach, to evaluate the airway liquid clearance of neonates. Our results indicate that the lung ultrasound score may be used to comprehensively and quantitatively characterize different lung regions and the process of lung liquid clearance of neonates in the first 24 h after birth. Our study also creates a quantitative comparison for future studies of predicting lung diseases such as neonatal respiratory distress syndrome and transient tachypnea of newborn. Lung ultrasound is a valuable tool for use in neonates.

## Figures and Tables

**Table 1:** Patients' clinical characteristics.

Variables	Patients(*n* = 66)
Gestational age (weeks days)	38^3/4^ (36^4/5^–39^2/3^)
Birth weight (g)	3355 (2860–3640)
Delivery mode	
Cesarean section	34
Vaginal delivery	32
Gender, female/male	31/35
Apgar at 1 minute	8 (8–9)
Apgar at 5 minutes	9 (9–9)

**Table 2:** Total lung ultrasound scores during the first 24 h.

Postnatal age at measurement, h	Median (upper and lower quartiles)
<3 h	8 (7–11)
6 h	2 (0–4)
24 h	1 (0–2)

## Data Availability

The data used to support the findings of this study are available from the corresponding author upon request.

## References

[1] Lichtenstein D. A., Mauriat P. (2012). Lung ultrasound in the critically ill neonate. *Current Pediatric Reviews*.

[2] Cattarossi L., Copetti R., Poskurica B. (2011). Radiation exposure early in life can be reduced by lung ultrasound. *Chest*.

[3] Zieleskiewicz L., Cornesse A., Hammad E. (2015). Implementation of lung ultrasound in polyvalent intensive care unit: impact on irradiation and medical cost. *Anaesthesia Critical Care & Pain Medicine*.

[4] Yousef N. (2016). L’échographie pulmonaire chez le nouveau-né. *Archives de Pédiatrie*.

[5] Bouhemad B., Brisson H., Le-Guen M., Arbelot C., Lu Q., Rouby J.-J. (2011). Bedside ultrasound assessment of positive end-expiratory pressure-induced lung recruitment. *American Journal of Respiratory and Critical Care Medicine*.

[6] Liu J. (2014). Lung ultrasonography for the diagnosis of neonatal lung disease. *The Journal of Maternal-Fetal & Neonatal Medicine*.

[7] Liu J., Chi J.-H., Ren X.-L. (2017). Lung ultrasonography to diagnose pneumothorax of the newborn. *The American Journal of Emergency Medicine*.

[8] Woods P. L. (2019). Utility of lung ultrasound scanning in neonatology. *Archives of Disease in Childhood*.

[9] Ren X.-L., Fu W., Liu J., Liu Y., Xia R.-M. (2017). Lung ultrasonography to diagnose pulmonary hemorrhage of the newborn. *The Journal of Maternal-Fetal & Neonatal Medicine*.

[10] Liu J., Cao H.-Y., Fu W. (2016). Lung ultrasonography to diagnose meconium aspiration syndrome of the newborn. *Journal of International Medical Research*.

[11] Li H., Li Y.-D., Zhu W.-W. (2018). A simplified ultrasound comet tail grading scoring to assess pulmonary congestion in patients with heart failure. *BioMed Research International*.

[12] Liu J., Copetti R., Sorantin E. (2019). Protocol and guidelines for point-of-care lung ultrasound in diagnosing neonatal pulmonary diseases based on international expert consensus. *Journal of Visualized Experiments*.

[13] Lissaman C., Kanjanauptom P., Ong C., Tessaro M., Long E., O’Brien A. (2019). Prospective observational study of point-of-care ultrasound for diagnosing pneumonia. *Archives of Disease in Childhood*.

[14] Adhikari S., Zeger W., Wadman M., Walker R., Lomneth C. (2014). Assessment of a human cadaver model for training emergency medicine residents in the ultrasound diagnosis of pneumothorax. *BioMed Research International*.

[15] Liu J., Cao H.-Y., Wang X.-L., Liao L.-J. (2016). The significance and the necessity of routinely performing lung ultrasound in the neonatal intensive care units. *The Journal of Maternal-Fetal & Neonatal Medicine*.

[16] Xiao J. S., Lee S., Do H. H., Oh K. H. (2017). Can limited education of lung ultrasound be conducted to medical students properly? A pilot study. *BioMed Research International*.

[17] Oh D. A., Kamlin C. O. F., Rogerson S. R. (2018). Lung ultrasound immediately after birth to describe normal neonatal transition: an observational study. *Archives of Disease in Childhood—Fetal and Neonatal Edition*.

[18] Blank D. A., Rogerson S. R., Kamlin C. O. F. (2017). Lung ultrasound during the initiation of breathing in healthy term and late preterm infants immediately after birth, a prospective, observational study. *Resuscitation*.

[19] Martelius L., Süvari L., Janér C. (2015). Lung ultrasound and static lung compliance during postnatal adaptation in healthy term infants. *Neonatology*.

[20] te Pas A. B., Davis P. G., Hooper S. B., Morley C. J. (2008). From liquid to air: breathing after birth. *The Journal of Pediatrics*.

[21] Morley Z., Gargani L., Adamicza Á. (2010). B-lines quantify the lung water content: a lung ultrasound versus lung gravimetry study in acute lung injury. *Ultrasound in Medicine & Biology*.

[22] Ma H., Huang D., Zhang M. (2015). Lung ultrasound is a reliable method for evaluating extravascular lung water volume in rodents. *BMC Anesthesiology*.

[23] Lichtenstein D., Mézière G., Biderman P., Gepner A., Barré O. (1997). The comet-tail artifact. *American Journal of Respiratory and Critical Care Medicine*.

[24] Targhetta R., Chavagneux R., Balmes P. (1994). Sonographic lung surface evaluation in pulmonary sarcoidosis: preliminary results. *Journal of Ultrasound in Medicine*.

[25] Caltabeloti F. P., Rouby J.-J. (2016). Lung ultrasound: a useful tool in the weaning process?. *Revista Brasileira de Terapia Intensiva*.

[26] Lichtenstein D. A. (2007). Ultrasound in the management of thoracic disease. *Critical Care Medicine*.

[27] Rouby J.-J., Arbelot C., Gao Y. (2018). Training for lung ultrasound score measurement in critically ill patients. *American Journal of Respiratory and Critical Care Medicine*.

[28] Wu J., Zhai J., Jiang H. (2015). Effect of change of mechanical ventilation position on the treatment of neonatal respiratory failure. *Cell Biochemistry and Biophysics*.

[29] Balaguer A., Escribano J., Roqué i Figuls M. (2006). Infant position in neonates receiving mechanical ventilation. *Cochrane Database of Systematic Reviews*.

[30] Kitchen M. J., Siew M. L., Wallace M. J. (2014). Changes in positive end-expiratory pressure alter the distribution of ventilation within the lung immediately after birth in newborn rabbits. *PLoS One*.

[31] Martelius L., Janér C., Süvari L. (2013). Delayed lung liquid absorption after cesarean section at term. *Neonatology*.

